# Local Dynamics of the Hydration Water and Poly(Methyl Methacrylate) Chains in PMMA Networks

**DOI:** 10.3389/fchem.2021.728738

**Published:** 2021-10-29

**Authors:** Yoshihisa Fujii, Taiki Tominaga, Daiki Murakami, Masaru Tanaka, Hideki Seto

**Affiliations:** ^1^ Department of Chemistry for Materials, Graduate School of Engineering, Mie University, Tsu, Japan; ^2^ Neutron Science and Technology Center, Comprehensive Research Organization for Science and Society, Tsuchiura, Japan; ^3^ Institute for Materials Chemistry and Engineering, Kyushu University, Fukuoka, Japan; ^4^ Institute of Materials Structure Science/J-PARC Center, High Energy Accelerator Research Organization, Tokai, Japan

**Keywords:** poly(methyl methacrylate), water, quasi-elastic neutron scattering, dynamic behavior, swelling

## Abstract

The dynamic behavior of water molecules and polymer chains in a hydrated poly(methyl methacrylate) (PMMA) matrix containing a small amount of water molecules was investigated. Water molecules have been widely recognized as plasticizers for activating the segmental motion of polymer chains owing to their ability to reduce the glass transition temperature. In this study, combined with judicious hydrogen/deuterium labeling, we conducted quasi-elastic neutron scattering (QENS) experiments on PMMA for its dry and hydrated states. Our results clearly indicate that the dynamics of hydrated polymer chains are accelerated, and that individual water molecules are slower than bulk water. It is therefore suggested that the hydration water affects the local motion of PMMA and activates the local relaxation process known as restricted rotation, which is widely accepted to be generally insensitive to changes in the microenvironment.

## Introduction

It is expected that the amount of polymer materials suitable for medical diagnosis and treatment will continue to increase in the coming years. To ensure such progress, the sorption and diffusion of water in polymers must be understood in detail, since they are important factors in drug delivery systems (Langer and Peppas, 1981; Arce et al., 2004), the desalination of water (Cath et al., 2006; Geise et al., 2010), fuel cells (Mauritz and Moore, 2004), coatings (van der Wel and Adan, 1999), and packaging (Auras et al., 2004). When a polymer is integrated as part of an organ, or as a diagnostic or *in situ* diagnostic or treatment equipment, the polymer surface is typically in contact with an aqueous phase, and so must be both biocompatible and able to adsorb water. Although poly(methyl methacrylate) (PMMA) is commonly used in many technological applications (Tetz and Jorgensen, 2015) due to its excellent cost performance in terms of its mechanical, optical, and interfacial properties, it does not exhibit a high biocompatibility or water content compared to other recently reported biocompatible materials. However, despite its low biocompatibility, PMMA has been employed as a medical material (Tetz and Jorgensen, 2015), although the accurate measurement of liquid water sorption in such vitreous polymers has been limited (Santos et al., 2017). Unlike transport experiments based on the use of polymers containing gases or vapors as diffusing agents, traditional weighing techniques that measure the diffusion of liquids in solid-state polymers (e.g., films) typically involve a tedious *ex situ* pat-and-weigh technique that can possess a low sensitivity and a high experimental error (Davis et al., 2011). In the case of PMMA/water mixtures, it was found that the polymer adsorbed only a small amount of water (∼2 wt%), which had little effect on the polymer biocompatibility.

In terms of the mechanical relaxation behavior of PMMA, Tanaka et al. (2008) studied the thermal relaxation of this polymer at the water interface by scanning force microscopy (Fujii et al., 2010) and demonstrated that the segmental motion could be released at room temperature. In their experiment, the penetration depth of the probe tip, which should correlate with the analytical depth, was ∼5 nm. Considering the existence of a PMMA density gradient close to the water interface, a faster molecular motion should appear closer to the interface. This phenomenon is primarily associated with the plasticization of the material, as extensively reported in the literature. Understanding how water molecules behave in a variety of restricted environments is therefore key to better understanding their roles in chemical and physical processes. In the context of biocompatibility, Tanaka and Mochizuki investigated the excellent blood compatibility of poly(2-methoxyethyl acrylate) (PMEA) in terms of its contact angle, equilibrium water content, and thermal analysis. Their differential scanning calorimetry (DSC) experiments showed that the water present in PMEA could be classified into three types, namely non-freezing water, freezing free water, and freezing intermediate water (Tanaka and Mochizuki, 2004). This categorization of water and its relationship with polymer biocompatibility have been verified using several experimental techniques (Miwa et al., 2009; Tanaka et al., 2021); however, the origin of intermediate water formation is not yet understood. As mentioned above, the majority of studies into the dynamics of hydrated polymer chains and the adsorbed water have been conducted on hydrophilic polymers that are easily swollen; few such studies have been carried out into hydrophobic polymer systems due to experimental difficulty.

Neutron scattering experiments on a protiated polymer sample can provide information regarding the self-correlation function of hydrogen atoms (H). Furthermore, since the difference between the incoherent cross-section of protium and the total cross-section of deuterium (D) is ∼80 times larger, the intensity scattered by a subset of the protium hydrogens can be strongly enhanced if the selective deuteration of the other hydrogens is possible by means of chemical methods. Thus, quasi-elastic neutron scattering (QENS) via back-scattering spectroscopy, which covers both the temporal picosecond to nanosecond scale and the spatial scale from 0.3 to 8 nm, is an effective means to investigate the dynamics of both hydration water and bulk water (Telling, 2020). In addition, through the use of selective deuteration, the self-correlated dynamics of a specific component of a complex system can be observed. To date, several QENS measurements have been performed to examine the dynamic behavior of hydration water in the vicinity of biocompatible materials (Colmenero and Arbe, 2013). For example, the authors investigated the dynamic behavior of hydration water molecules between phospholipid membranes and demonstrated the existence of three types of water (Yamada et al., 2017; Seto and Yamada, 2020).

In this article, we report QENS results for mixtures of water and PMMA carried out from low to physiological temperature to estimate the activation energy of the local motion of PMMA. The hydration water in the vicinity of the PMMA chains can be categorized into three types: slow water with a relaxation time of more than sub-nanoseconds, medium-speed water whose relaxation time is slower than that of the bulk water, and fast water, whose characteristics are similar to that of bulk water. Although it is known that the dynamic behavior of medium-speed water is closely related to changes in the local motion of PMMA (Higgins and Benoit, 1994), the detailed dynamics of hydrated water and the local motion of PMMA, the latter of which is expected to change due to hydration, have not yet been clarified. Therefore, the correlation between the water and PMMA dynamics in the hydrated state are elucidated using the H/D contrast in both the water and PMMA components.

## Experimental

**FIGURE 1:**
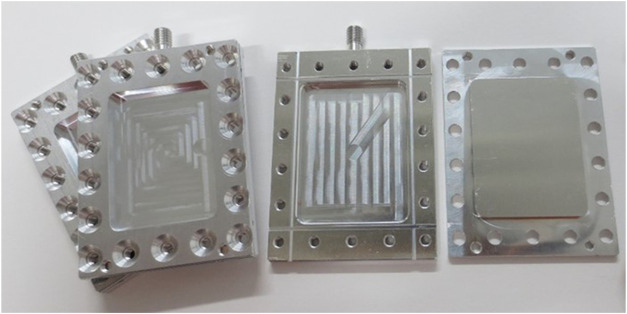
Photographic image of the flat aluminum cell used in this study. The PMMA sheet wrapped in Nb foil was installed in the center of the sample space (30 mm × 40 mm) and sealed by an indium wire to prevent the evaporation of water during measurement.

The perdeuterated poly(methyl methacrylate) (dPMMA) was purchased from Polymer Source Inc., with a number-average molecular weight (*M*_n_) of 15,500 g/mol, a weight average molecular weight (*M*_w_) of 16,000 g/mol, and an *M*_w_/*M*_n_ ratio of 1.02. Protiated PMMA (hPMMA) was also purchased from Polymer Source Inc., with an *M*_n_ value of 15,000 and an *M*_w_/*M*_n_ ratio of 1.12. These polymers were used as received without any further purification. The dPMMA was dissolved in perdeuterated toluene to prevent the exchange of D and H during film preparation. Similarly, the hPMMA film was prepared in a protiated toluene solution. The film thicknesses were controlled to ∼1 and 0.2 mm, respectively, to maintain a neutron transmittance of ∼90% for each sample. The films were annealed under vacuum at 150°C for 24 h. Hydration films were prepared by immersing in Milli-Q water (H_2_O) and deuterated water (D_2_O, ≥99.96 atom% D) purchased from Sigma-Aldrich Co. LLC. The water content was calculated using the weight differences for the samples before and after water absorption. The water content of dPMMA/H_2_O was 2.7 wt%, while that of dPMMA/D_2_O was 2.4 wt%; these values are comparable to the saturated water content of PMMA. Since PMMA film is brittle even when it contains water, a flat specimen of the film was used since it cannot be bent into the cylindrical sample shape that is required for the QENS measurement cell. Thus, the sample sheets were installed on flat aluminum cells, as shown in Figure 1. The samples were wrapped with Nb foil with a thickness of 25 μm to avoid corrosion of the aluminum upon contact with water. Due to the fact that the scattering cross-sections of aluminum and the Nb foils are small in comparison to the incoherent cross-section of protons in the sample, their influence can be ignored by minimizing the thicknesses of the Nb and Al components.

The QENS measurements were performed using a time-of-flight near-backscattering spectrometer BL02 (DNA) (Shibata et al., 2015; Seto et al., 2017; Kajimoto et al., 2019) at the Materials and Life Science Experimental Facility (MLF) at the Japan Proton Accelerator Research Complex (J-PARC). The injected proton beam power incident on the neutron target was approximately 500 kW. The energy resolution was 12 μeV (high-flux mode), and was achieved using a Si 111 analyzer. The flat plate sample was placed at an angle of 30° with respect to the incident beam, and data were acquired in the *Q* range of 0.125–1.54 A^−1^. The QENS measurements were performed at −30, 5, and 37°C, with energy transfer (*E*) ranges of −0.2 < *E* [meV] < 1.0. Data analysis was performed without instrumental background or sample cell scattering. The detector efficiency and instrumental resolution function were obtained from the incoherent scattering of the vanadium standard. The dynamics of PMMA in the dry and hydrated states were measured using hPMMA and D_2_O. When evaluating the dynamics of the water molecules present within the PMMA matrix, fitting was performed after subtracting the result of (dPMMA/D_2_O) from (dPMMA/H_2_O) at 37°C. This subtraction operation reduces the contribution of scattering from dPMMA to the maximum possible extent, and allows a detailed discussion of the water dynamics.

DSC measurements were carried out using an EXSTAR X-DSC7000 (Hitachi High-Tech Corp.) instrument. During scanning, the samples were cooled from 30 to −100°C at a rate of 5°C/min, held at −100°C for 5 min, and heated to 30°C at a rate of 5°C/min under a flow of nitrogen.

## Results and Discussion

**FIGURE 2:**
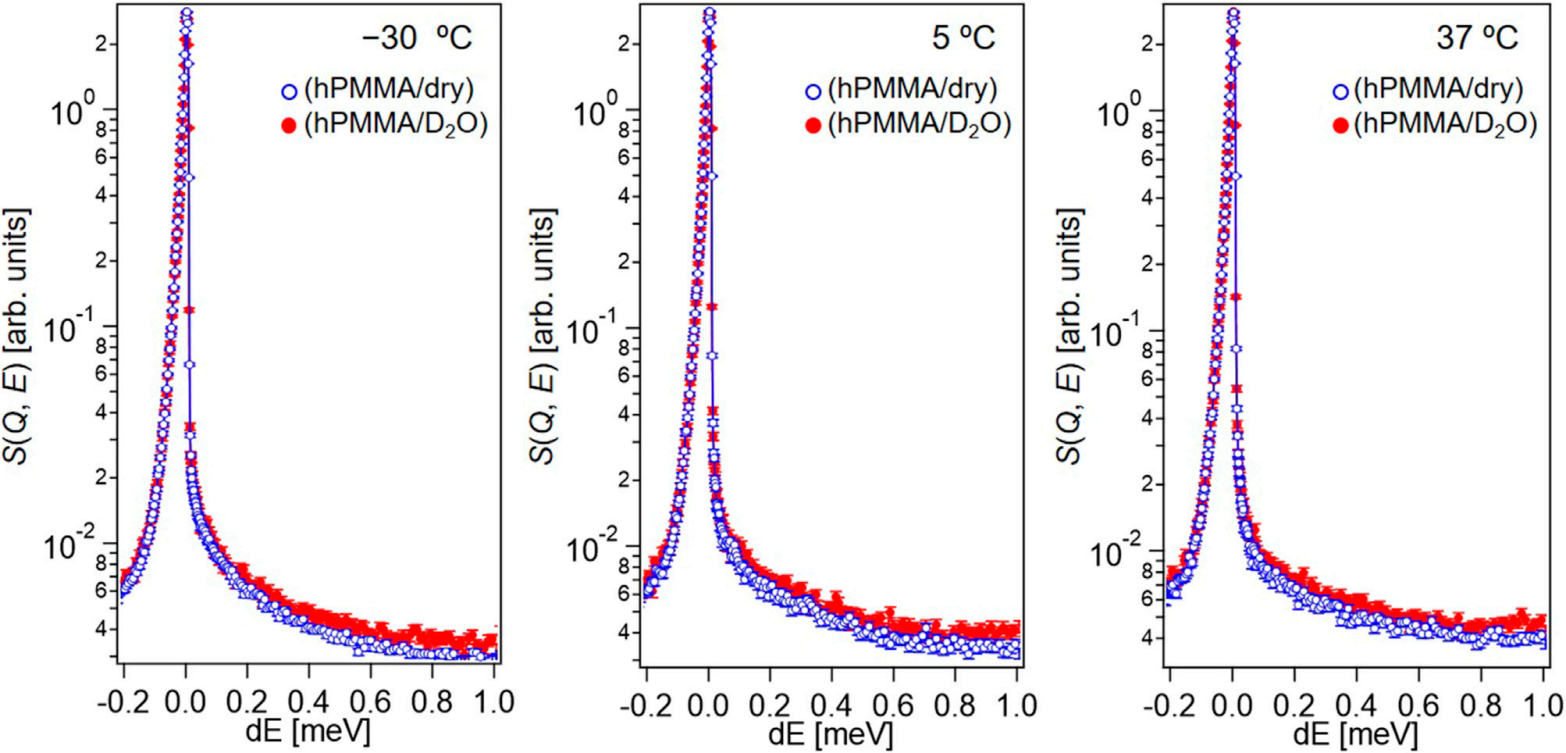
QENS profiles of hPMMA/D_2_O (red closed circles) and dry hPMMA (blue open circles) at *Q* ∼1 Å^−1^ of in the high-flux mode at −30, 5, and 37°C.

To investigate the molecular dynamics of PMMA in the dry and hydrated states, dry hPMMA and hPMMA/D_2_O were prepared. During the QENS measurements of the dry hPMMA and hPMMA/D_2_O samples, protons were only included in PMMA, so that the QENS signals mainly originated from the dynamics of the PMMA chains. Figure 2 shows the QENS profiles of these two samples at *Q* ∼1 Å^−1^, which were recorded at −30, 5, and 37°C.

**FIGURE 3:**
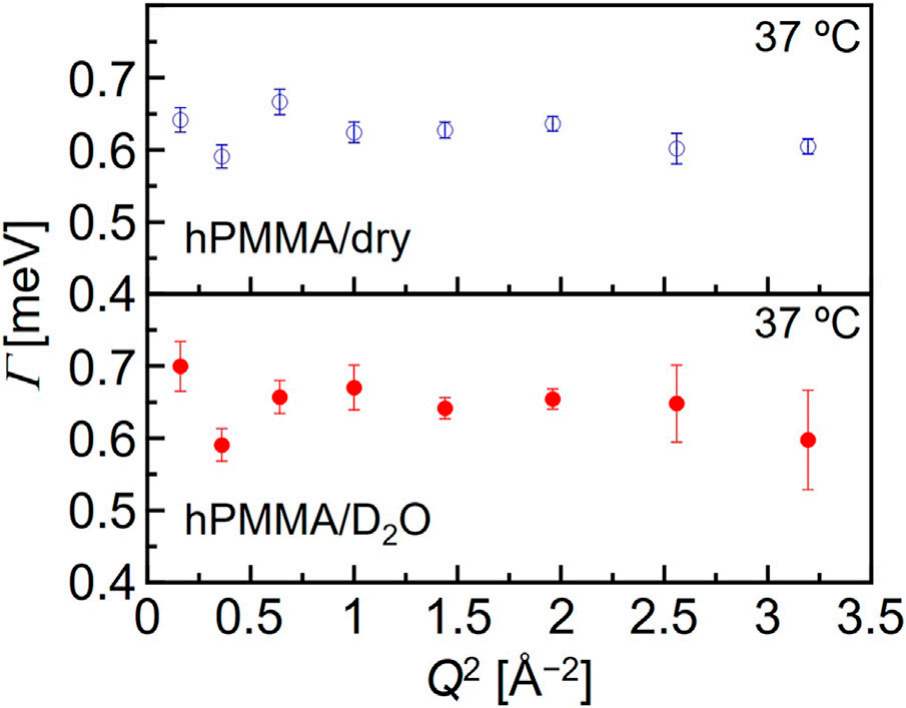
Typical *Q*-dependence of the Lorentz function half width at half maximum (*Γ*) for the dry hPMMA and the hydrated hPMMA at 37°C.

Upon examination of Figure 2, it is clear that the QENS profiles from the hydrated hPMMA sample (red closed circles) were broader than those from the dry hPMMA sample (blue open circles) at all temperatures measured. This indicates that the molecular mobility of PMMA was activated in the presence of water under these conditions, even when the temperature was lower than that required for the beta-relaxation process related to the side chain (i.e., -COOCH_3_) of PMMA (McCrum et al., 1967). We then examined the assignment of the observed molecular motion by evaluating the temperature dependence of peak broadening (*Γ*). More specifically, we employed the sum of two terms, namely the delta function and the Lorentz function, to interpret the QENS profiles as follows:

$$S\left(Q,E\right)=R\left(Q,E\right)⊗\left(v_{1}\delta \left(Q,E\right)+v_{2}L\left(\Gamma ,E\right)\right)+B_{g,}$$ (1)

where *R*(*Q*, *E*), *δ*(*Q*, *E*), *L*(*Γ*, E), and *B*_g_ represent the resolution function, the delta function, the Lorentz function, and the constant background, respectively. In addition, the *ν*_*n*_ (*n* = 1,2) is related to the number of hydrogen atoms in each motion. Here, *Γ* is the half-width at half-maximum (HWHM) of the Lorentz function. It is reasonable to interpret that *δ*(*E*) and *L*(*Γ*, E) represent the immobile (frozen) chains and the mobile functional groups, respectively, which can be obtained by the resolution and the *Q*-*E* window of the DNA spectrometer in the high-flux mode. Fitting was performed using the least-squares method on Igor Pro (WaveMetrics). This interpretation is supported by the weak *Q*-dependence of *Γ* (shown in Figure 3), which could be evidence that the Lorentz function originates from a local motion of PMMA chains.

**FIGURE 4:**
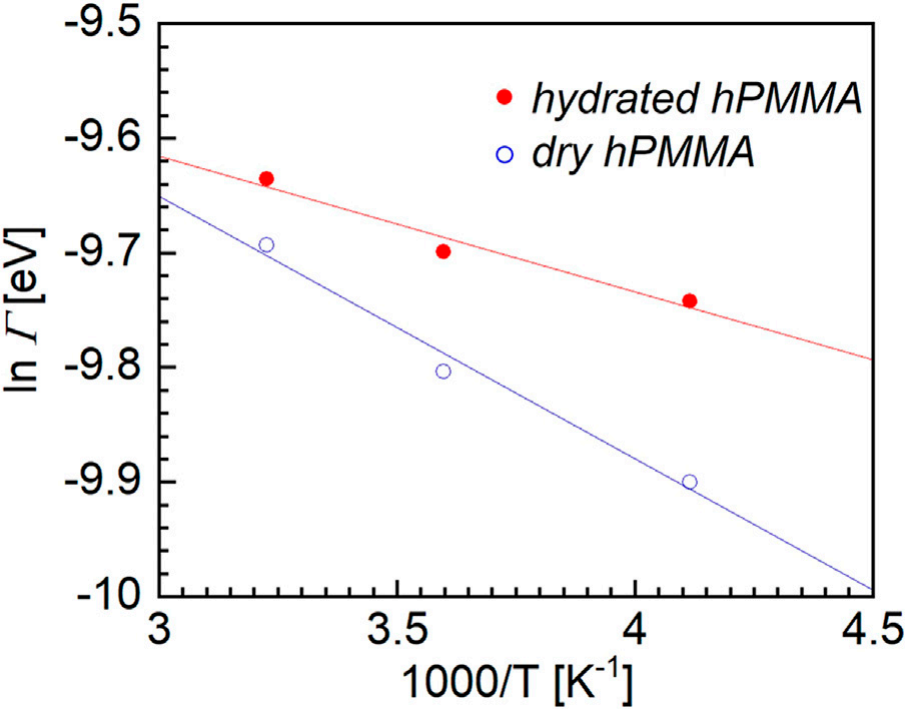
Semilogarithmic plot between the half width at half maximum (*Γ*) of the Lorentz function, versus the reciprocal of the absolute temperature for the dry hPMMA and hydrated hPMMA samples.

Figure 4 shows semilogarithmic plot for the dry and hydrated hPMMA samples. The horizontal axis represents the reciprocal of the measurement temperature (1/*T*), while the ordinate is the logarithm of the *Γ* of the Lorentz function. As can be seen from this figure, the HWHMs of the Lorentz function for both the dry and hydrated hPMMA samples increased with increasing temperature. Within the temperature range employed, the relationship between ln *Γ* and *T* ^−1^ seems to be linear, thereby indicating that the plots can be represented using an Arrhenius type equation. This is a characteristic feature for relaxation processes in confined systems, such as in the case of side-chain rotation. Subsequently, using Eq. 2, the apparent activation energy (ΔH*) was obtained for the local molecular motion:

$$\Gamma =\Gamma_{\infty }\exp\left(-\Delta H*/k_{B}\cdot T\right)$$ (2)

In this equation, ΔH* is the activation energy barrier for rotation, *k*_B_ is the Boltzmann constant, and *Γ*_∞_ is a temperature-independent pre-exponential factor. Based on previous literature (Mukhopadhyay et al., 1998), a value of 4.8 meV was adopted as *Γ*_∞_ in the case of PMMA.

The activation energies of the dry and hydrated hPMMA samples were then estimated from the plots presented in Figure 4. More specifically, the ΔH* value for the hydrated hPMMA was estimated to be 3.1 kJ/mol, which was significantly lower than the corresponding value for the dry hPMMA, i.e., 5.1 kJ/mol. It was therefore considered that the local motion observed in the QENS measurement originates from the CH_3_ moieties bind to the polymer side-chain and backbone since the measurement temperature range was well below the glass transition temperature at which movement of the main chain was frozen. From the magnitude of these values of ΔH*, the molecular dynamics observed during these QENS measurements were attributed to the local rotational motion of PMMA, which corresponds well with previous studies of QENS measurements (Arrighi et al., 1995; Sakai and Arbe, 2009). This difference could be attributed to the fact that the local molecular motion in the hydrated sample is accelerated by water molecules. It should be noted here that early studies into the rotational motion of the ester methyl group in PMMA showed a peculiar temperature dependence for the width of the quasi-elastic component (Kunal et al., 2008), wherein the full width at half maximum (FWHM) versus the inverse of temperature did not follow a simple Arrhenius law. More specifically, the apparent activation energy was measured, and was found to vary from 1 kJ/mol at 150 K to 7 kJ/mol at room temperature. It should be emphasized that molecular motion on a relatively small scale is generally insensitive to changes in the microenvironment (McCrum et al., 1967). For polymers, these can arise from the motion of pendant groups and usually have no relation to structural relaxation or to the surroundings. On the other hand, a recent study using two-dimensional nuclear magnetic resonance spectroscopy revealed that β-relaxation is a complex process consisting of the hindered rotation of side chain groups in addition to local cooperative movement (Schmidt-Rohr et al., 1994). Furthermore, Fukao et al. (2001) used dielectric relaxation spectroscopy to examine the α_a_- and β-relaxation processes in ultrathin films of PMMA. They claimed that once the thickness became smaller than a critical value of ∼100 nm, the relaxation temperatures for the α_a_- and β-processes decreased with a reduced thickness owing to the surface effects. This indicates that the β-process depends on the microenvironmental changes. Importantly, our findings do not contradict their results, and may suggest that extremely small-scale molecular motions, such as those of methyl groups, are also accelerated by water molecules.

It is known that characteristic relaxation times can vary significantly depending on the local configuration around the relaxing unit. For example, movement of the methyl group can be inhibited by direct connection to the main chain. However, if the methyl group exists as part of an ester or ether group attached to the main chain, as in the case of PMMA, these librational modes are hardly observable (Mukhopadhyay et al., 1998). Therefore, further QENS experiments using partially deuterated PMMA systems should be performed to clarify whether the local motion originates from directly linked methyl groups or from ester methyl groups.

**FIGURE 5:**
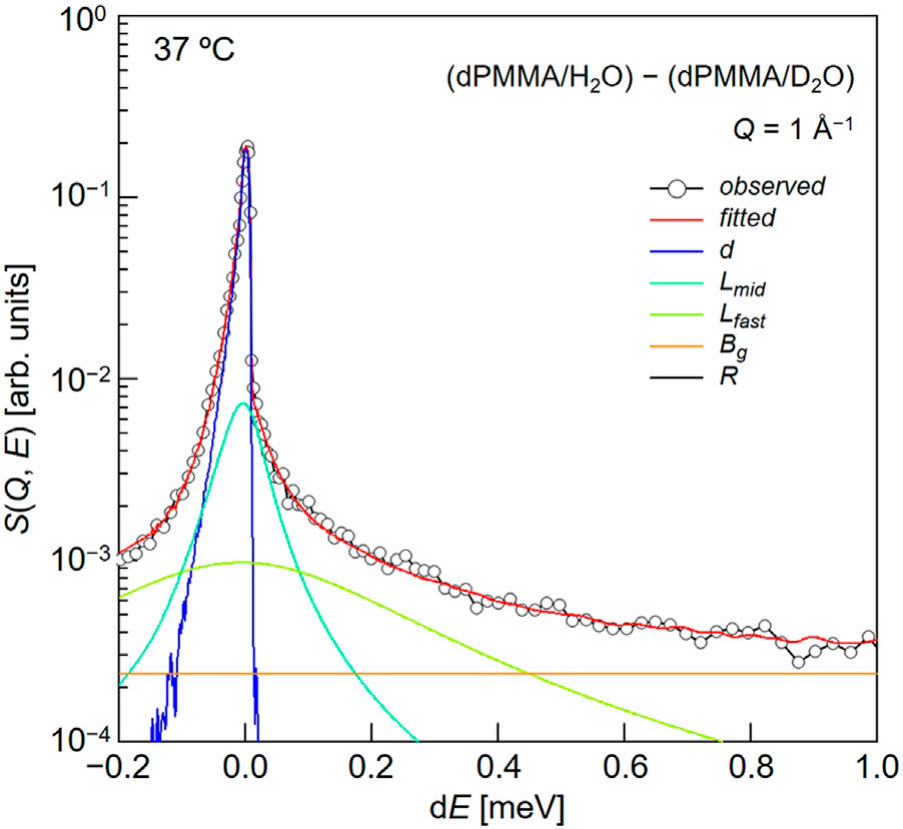
Typical fitting result for a QENS profile obtained from the difference between dPMMA/H_2_O and dPMMA/D_2_O.

Subsequently, we evaluated the dynamical behavior of water in the vicinity of the polymer chains in the hydrated PMMA. To evaluate the motion of the hydration water molecules present within the PMMA matrix, QENS experiments were carried out on the mixtures of dPMMA/H_2_O and dPMMA/D_2_O at 37°C, and the data corresponding to dPMMA/D_2_O were subtracted from those of dPMMA/H_2_O to estimate the contribution from H_2_O. The QENS profiles were then obtained from the result of subtraction at *Q* ∼1 Å^−1^, as shown in Figure 5. For this purpose, we used the sum of three terms, namely one delta function and two Lorentz functions, to interpret the QENS profiles as follows:

$$S\left(Q,E\right)=R\left(Q,E\right)⊗\left(v_{1}\delta \left(Q,E\right)+v_{2}L_{\text{mid}}\left(\Gamma_{\text{mid}},E\right)+v_{3}L_{\text{fast}}\left(\Gamma_{\text{fast}},E\right)\right)+B_{g}$$ (3)

In this equation, the delta function represents the slow mode (i.e., narrower than the instrumental resolution) component, whereas the subscripts “mid” and “fast” indicate the middle-speed water and fast water components, respectively.

**FIGURE 6:**
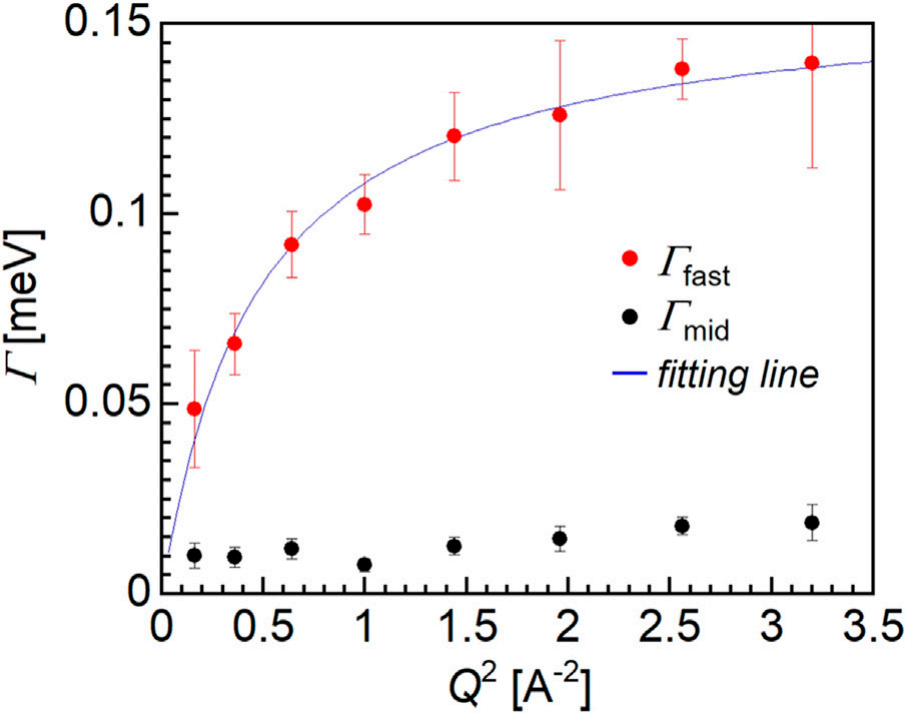
*Q* ^2^-dependence of HWHM (*Γ*).

Figure 6 shows the *Q-*dependence of *Γ*_mid_ (black closed circles) and *Γ*_fast_ (red closed circles), and based on the observed trend, only *Γ*_fast_ could be explained by the jump diffusion model shown as the fitting line (blue). The fitting assuming the jump diffusion model (Bée, 1988) was performed based on the following equation:

$$\Gamma =Dq^{2}/\left(1+Dq^{2}\tau \right)$$ (4)

where *τ* and *D* are the mean residence time and the diffusion coefficient, respectively. Thus, from the fitting of the *Q-*dependence of *Γ*_fast_, the *τ* and *D* values for the water molecules could be estimated. The diffusion coefficient of the fast mode was determined to be 1.3 × 10^–9^ m^2^/s, which is approximately half of the value for bulk water, i.e., 2 × 10^–9^ m^2^/s. Moreover, the mean residence time was 2.9 × 10^–11^ s for the fast mode water molecules, which is more than three times smaller than corresponding value for the bulk water molecules (i.e., 8 × 10^–12^ s). It should be noted that the motion of the hydration water molecules within the PMMA matrix is slower compared to that of the bulk water, despite PMMA being a hydrophobic polymer.

The relaxation time of the fast mode water ranges from approximately 10^–12^ to 10^–11^ s, which is one or two orders of magnitude faster than the NMR correlation time for intermediate water. It should be noted that the motion of water molecules observed by QENS was characterized by jump diffusion while NMR measurements mainly observe molecular rotations. Additionally, the diffusion coefficient of the fast mode water determined herein is comparable to that of the free water molecules in the case of a mixture of phospholipid (Seto and Yamada, 2020). This result therefore confirmed that water exists in the form of intermediate water in the hydrated PMMA, and this finding can be explained by considering the formation of hydrogen bonds between the water molecules and the carbonyl groups of PMMA (Lee et al., 2010).

**FIGURE 7:**
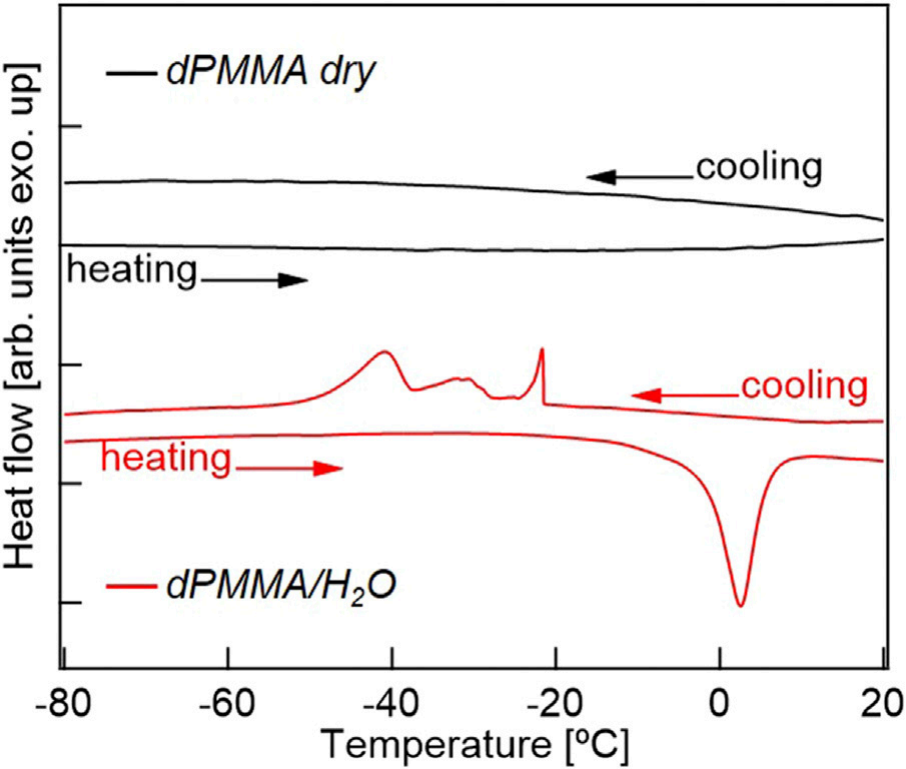
DSC thermograms for dPMMA in its dry and hydrated states.

Previously, the intermediate water present in biocompatible polymers has been mainly discussed based on DSC measurements, and was only found in polymers with relatively high water contents. Thus, Figure 7 shows the DSC cooling and heating profiles of the dry dPMMA and hydrated dPMMA samples examined herein. As shown in the cooling profiles, a significant difference could be seen between the thermograms of the dry and hydrated PMMA specimens. More specifically, in the hydrated PMMA, exothermic peaks were observed in the range of −20 to −45°C; these peaks were not observed in the case of the dry PMMA. Based on previous results (Tanaka and Mochizuki, 2004), it can be considered that these exothermic peaks are caused by the freezing of hydration water. In addition, compared with previously reported results for biocompatible materials with high water contents, the peak at approximately −20°C originates from the freezing of free water, while the peak at approximately −40°C may be derived from the intermediate water. The wide range of exothermic peaks in the cooling scan may reflect the difference in the hydration state due to the heterogeneity of the hydration water in PMMA. The proportion of intermediate water to the total amount of water was calculated from the enthalpy of water crystallization obtained from the DSC thermograms with reference to the literature (Toyokawa et al., 2021). Assuming that the peak at approximately −45°C was derived from intermediate water, 28% of the 2.6 wt% water content of PMMA is present as intermediate water, which is an extremely small value compared to those determined for biocompatible materials, such as PMEA (Tanaka and Mochizuki, 2004). In a previous review by Tsuruta, it was mentioned that the shape change of the time-resolved IR spectrum (in the OH stretching vibrational region) for the sorbed water of PMMA was surprisingly similar to that of PMEA despite the small amount of sorbed water (Tsuruta, 2010). However, considering the supportive research carried out to date and the results obtained in the current study, our findings represent the first observation of intermediate water in a hydrophobic polymer with a low water content. In addition, the discovery that local restricted motions are affected by extremely small amounts of hydrated water molecules also adds new knowledge to this area.

## Conclusion

In this work, we focused on the dynamic behavior of water in the vicinity of poly(methyl methacrylate) (PMMA), which is a typical glassy polymer with a low water solubility. For this purpose, quasi-elastic neutron scattering (QENS) experiments were carried out on the dry and hydrated states of PMMA in combination with judicious hydrogen/deuterium labeling to independently investigate the dynamic behaviors of the polymer chains and the hydration water. We found that the motion of the hydration water molecules in PMMA was slower than that in the bulk water, despite PMMA being a hydrophobic polymer. Based on the obtained results, the cause of the weak biocompatibility of PMMA, a hydrophobic polymer with a low water content, was clarified. These results are also of interest since the majority of studies carried out into the dynamics of hydrated polymer chains and the adsorbed water have been conducted on hydrophilic polymers that are easily swollen, with very few reports being published into hydrophobic polymers.

## Data Availability

The original contributions presented in the study are included in the article/Supplementary Material, further inquiries can be directed to the corresponding authors.
